# Complete Genome Sequence of Listeria monocytogenes NTSN, a Serovar 4b and Animal Source Strain

**DOI:** 10.1128/genomeA.01403-14

**Published:** 2015-01-15

**Authors:** Weijun Tan, Guoliang Wang, Zhiming Pan, Yuelan Yin, Xin’an Jiao

**Affiliations:** Jiangsu Key Laboratory of Zoonosis, Jiangsu Co-Innovation Center for Prevention and Control of Important Animal Infectious Diseases and Zoonoses, Yangzhou University, Yangzhou, China

## Abstract

Listeria monocytogenes is an important foodborne pathogen that causes infections in humans and animals and has a high mortality rate. The complete genome sequence of L. monocytogenes strain NTSN, a highly virulent and serovar 4b strain isolated from the brains of sheep in Jiangsu Province, China, is presented here.

## GENOME ANNOUNCEMENT

Listeria monocytogenes is a Gram-positive facultative intracellular foodborne pathogen causing severe diseases, such as septicemia, abortion, meningitis, and even death, in humans and animals (1, 2). More than 98% of human Listeria infections are caused by L. monocytogenes serovar 1/2a, 1/2 b, 1/2c, and 4b strains (3). During an outbreak of listeriosis, L. monocytogenes primarily causes gastroenteritis in healthy individuals and more severe symptoms in immunocompromised people, no matter whether newborn babies or the elderly (4). To date, L. monocytogenes strains can be divided into four evolutionary lineages and 13 serotypes with different pathogenicities; therefore, it is an important model for studying intracellular parasitism and its pathogenic mechanism (5, 6). Here, we determined the complete genome sequence of L. monocytogenes strain NTSN, a highly virulent and serovar 4b strain isolated from the brains of sheep in Jiangsu Province, China (7).

The nucleotide sequence was sequenced using a 454 Genome Sequencer FLX. In total, 174,636 reads, composed of 84,402,981 bp, were obtained (~29-fold genome coverage) and assembled with 454 software Newbler version 2.3, generating a total of 20 contigs. After gap closing through primer walking and sequencing of the PCR products, we finally obtained the complete circular NTSN genome, consisting of 2,904,500 bp, with a G+C composition of 38.0%.

The putative protein-coding sequences (CDSs) were based on the prediction of Glimmer 3.02 (8) and ZCURVE 2.0 (9) and searched against the NCBI nonredundant (NR) databases and the Kyoto Encyclopedia of Genes and Genomes (KEGG) to acquire the functional annotation. The tRNA and rRNA genes were directly identified using tRNAscan-SE (10) and RNAmmer (11), respectively. The genome sequence of NTSN contains 2,821 protein-coding genes, with an average length of 304 amino acids, six 16S-5S-23S operons, and 67 tRNA genes, similar to other L. monocytogenes strains, and it harbors no plasmid. The genome sequences of L. monocytogenes 4b F2365 (GenBank accession no. NC_002973) and 4b LL195 (GenBank accession no. NC_019556) were obtained from GenBank as references. Synteny is highly conserved among the three genomes. Furthermore, all of them belong to sequence type 1 (ST1) (*abcZ-3*, *bglA-1*, *cat-1*, *dapE-1*, *dat-3*, *ldh-1*, and *lhkA-1*) *in silico* multilocus sequence typing (MLST) analysis, which shows a high similarity. A special analytical report regarding the genome of this strain will be included in a future publication.

### Nucleotide sequence accession number.

The complete genome sequence of strain NTSN has been deposited in GenBank under accession no. CP009897.
